# Periodontal Health in Individuals Living with HIV: An Exploratory and Descriptive Molecular Approach of Microbial Interspecific and Intraspecific Diversity in Brazilian Patients

**DOI:** 10.3390/microorganisms13040867

**Published:** 2025-04-10

**Authors:** Patricia N. Olivares Ponce, Lana Bitencourt Chaves, Daiana de Souza Perce-da-Silva, Ana Luiza Carneiro-Alencar, Cinthia Magalhães Rodolphi, Isabela Ferreira Soares, Rodrigo Nunes Rodrigues-da-Silva, Ana Caroline Alves-da-Silva, Fabio Vidal Marques, Rafael Vidal Peres, Dennis de Carvalho Ferreira, Rodrigo Carvalho de Souza, Cristiane Gonçalves, Lucio Souza Gonçalves, Josué da Costa Lima-Junior

**Affiliations:** 1 Immunoparasitology Laboratory, Oswaldo Cruz Institute, Fiocruz, Rio de Janeiro 21040-360, RJ, Brazil; 2 Postgraduate Program in Dentistry, Faculty of Dentistry, Estácio de Sá University-IDOMED, Rio de Janeiro 22640-100, RJ, Brazil; 3 Clinical Immunology Laboratory, Oswaldo Cruz Institute, Fiocruz, Rio de Janeiro 21040-360, RJ, Brazil; 4 Basic and Applied Immunology Laboratory, Petrópolis Medical School, Arthur Sá Earp Neto University Center, Petrópolis 25680-120, RJ, Brazil; 5 Laboratory of Hantaviroses and Rickettsioses, Oswaldo Cruz Institute, Fiocruz, Rio de Janeiro 21040-360, RJ, Brazil; 6 Faculty of Dentistry, Rio de Janeiro State University, Rio de Janeiro 20551-030, RJ, Brazil

**Keywords:** HIV, oral health, bacterial diversity, molecular identification, genetic diversity

## Abstract

Oral manifestations of HIV infection can be an early sign of the disease and may indicate progression to AIDS. Although antiretroviral therapies, especially highly active antiretroviral therapy (HAART), have reduced the prevalence of HIV-related oral lesions, ongoing updates in diagnosis and treatment are essential due to the extended life expectancy of individuals living with HIV. Periodontal disease is a significant concern in these patients, influenced by altered immune responses and microbial dynamics, though the mechanisms are not fully understood. This exploratory study aimed to investigate the oral microbiota and periodontal disease prevalence in HIV-positive individuals by analyzing subgingival plaque samples from 24 patients. We identified 12 bacterial species using Polymerase Chain Reaction (PCR) and amplicon sequencing. Seven species were detected, with *Filifactor alocis*, *Tannerella forsythia*, and *Porphyromonas endodontalis* being the most common. *Porphyromonas gingivalis* was present in only 13.6% of samples, while *T. forsythia* was found in 58.3%. Genetic diversity was also observed in *P. endodontalis* and *Selenomonas sputigena* amplicons, with specific single nucleotide polymorphisms (SNPs) identified in both species. These results highlight the complex microbial interactions in the oral environments of people living with HIV, emphasizing the need for personalized diagnostic and therapeutic strategies for managing oral health in this population.

## 1. Introduction

Oral health in individuals living with HIV (PLHIV) poses unique challenges and complexities. While HAART has significantly improved the overall health outcomes of PLHIV, oral manifestations remain a significant concern, impacting both diagnosis and management strategies [1]. Candidiasis, particularly oral candidiasis, stands as a hallmark of HIV-related oral lesions, with various clinical presentations prevalent among PLHIV. Additionally, periodontal disease presents a significant burden, characterized by altered microbial profiles and immune responses in PLHIV [2].

The oral microbiota plays a crucial role in the health and disease of individuals infected with HIV, with alterations observed in both diversity and composition, especially following HAART. While studies suggest a reduced microbial diversity in HIV-infected individuals, particularly post-HAART, further research with larger sample sizes is warranted to elucidate the potential impact of HIV infection and treatment on the oral microbiome. Moreover, understanding these microbial alterations may shed light on the emergence of opportunistic pathogens in the oral cavity.

In this scenario, periodontal disease in PLHIV presents a complex interplay of microbial colonization and host immune responses. While classic periodontal pathogens are prevalent in both HIV-positive and -negative individuals, PLHIV may exhibit a predisposition to additional infections by atypical microorganisms. Notably, the immunosuppressive state in PLHIV may favor the colonization of such pathogens, contributing to the severity of periodontal disease. In addition to considering the classic pathogens, the intraspecific diversity of periodontal bacteria within PLHIV warrants attention. Variations in the genotypic and phenotypic characteristics of periodontal pathogens may influence disease progression and treatment outcomes in this population [3]. Understanding the intraspecific diversity of these bacteria can provide observations into the pathogenicity and virulence factors associated with periodontal disease in PLHIV, ultimately guiding more effective therapeutic interventions.

Advancements in molecular methods, particularly Polymerase Chain Reaction (PCR) and sequencing techniques, have revolutionized microbial identification, allowing for the detection of unculturable or difficult-to-culture species. These methods have significantly contributed to our understanding of microbial dynamics in oral diseases, offering more specificity into exploring previously unknown pathogens and enhancing diagnostic accuracy. One of the key tools in this process is the analysis of the 16S ribosomal RNA (rRNA) gene, which provides a powerful means of characterizing the bacterial composition of the oral microbiome [4]. Through the sequencing of the 16S rRNA gene, clinical and researchers have been able to gain deeper access to the global genetic, metabolic, and ecological changes associated with periodontitis [4]. This approach has revealed that the disease microbiome is enriched in virulence factors and adapted to a parasitic lifestyle that takes advantage of the disrupted host homeostasis [4]. Moreover, the disease state has been found to occupy a narrow region within the space of possible configurations of the oral microbiome, suggesting a common structure that is distinct from completely healthy samples [4]. Ultimately, the 16S bacterial analysis has emerged as a crucial tool in the diagnosis and identification of periodontal and peri-implant bacterial species, enabling a deeper understanding of these complex microbial communities and their role in oral health and disease [4,5]. In patients with HIV, 16S analysis has been used to identify shifts in the oral microbiome associated with disease progression and immune status [6]. This highlights the broad applicability of this approach in understanding the oral microbiome and its clinical implications.

Studies utilizing the 16S rRNA gene as a molecular marker have highlighted the significance of intraspecific diversity within bacterial communities, including those in the oral cavity [7]. This diversity has been implicated in influencing the clinical presentation and progression of periodontal diseases, with specific bacterial variants or strains potentially correlating with disease severity and treatment responses. Critically, the impact of periodontal disease may be exacerbated in immunocompromised individuals such as PLHIV [8]. In these cases, dysbiotic shifts in the oral microbiome could intensify clinical manifestations and compromise treatment outcomes.

Understanding intraspecific diversity within the 16S rRNA gene of oral bacteria and its implications for the pathogenesis and clinical course of periodontal diseases holds significant promise for personalized diagnosis and targeted therapeutic interventions, particularly in immunocompromised patients [4,9]. Moreover, addressing the knowledge gap in intraspecific microbial diversity among clinical samples from South American countries is crucial. Exploring such diversity could unveil regional variations in the oral microbiome and their associations with periodontal health outcomes, thus informing the development of more effective prevention and treatment strategies tailored to individual patient needs. Therefore, we aimed to investigate the microbial diversity present in Brazilian patients with HIV by employing molecular techniques, focusing on both the interspecific (between different microbial species) and intraspecific (within the same microbial species) variations observed in microbial communities. Ultimately, this research will enhance our understanding of microbial diversity’s role in health and disease, with implications for developing targeted therapeutic strategies and improving patient outcomes.

## 2. Materials and Methods

### 2.1. Study Population and Sample Collection

The study involved 24 patients enrolled in an ongoing HIV infection research project at the Department of Oral Pathology and Diagnosis, School of Dentistry, Universidade Estácio de Sá. After providing informed consent and receiving comprehensive information about the study’s objectives, risks, and benefits, participants were included if they were patients with HIV/AIDS aged over 13 years. Patients with incomplete HIV status data in their medical records were excluded.

Participants’ socio-demographic information, including smoking status, age, and gender, as well as their medical history—such as diabetes, hypertension, heart disease, and use of ARV drugs—was gathered through a self-administered questionnaire. HIV-related data, including details on antiretroviral therapy, T-CD4 + lymphocyte counts, and viral load, were extracted from medical charts.

Subgingival plaque samples were collected using a sterile curette, which was scraped along the tooth at the gingival sulcus. The sampling was conducted under aseptic conditions, with samples collected into 0.5 mL of sterile Tris/EDTA buffer. No bleeding was observed at the sampling sites during collection. The collected plaque samples were subsequently used for molecular analysis to evaluate the diversity of 12 bacterial species.

### 2.2. Clinical Record

Periodontal measurements were assessed during a complete periodontal examination performed at the School of Dentistry, at the Estácio de Sá University. They were recorded at six sites per tooth (distobuccal, buccal, mesiobuccal, distolingual, lingual, and mesiolingual) in all teeth, excluding third molars. The examination was performed by the same experienced periodontist observer (intraclass correlation coefficient of 0.86 for probing depth and 0.80 for clinical attachment loss). Screening of the clinical assessments was evaluated (periodontal probing depth (PPD) (mm), clinical attachment loss (CAL) (mm), bleeding on probing (BOP) [10] and visible supragingival biofilm (VSB) [11]) using a periodontal probe with a diameter of 0.5 mm (PCV12, HuFriedy, Chicago, IL, USA).

### 2.3. DNA Extraction

DNA extraction followed the protocol of the QIAamp^®^ DNA Blood Mini Kit (Qiagen, Hilden, Germany). Initially, lyophilized Protease K was reconstituted and all kit components were prepared. Subsequently, 20 µL of Protease K and 200 µL of the sample were combined in a 1.5 mL Eppendorf tube with 200 µL of Buffer AL. After vortexing for 15 s, the tube was incubated at 56 °C for 10 min. Next, 200 µL of 96–100% ethanol was added, vortexed for 15 s, and the lysate was transferred to a column without touching the column walls, using an attached Eppendorf tube. The column was centrifuged at 8000 rpm for 3 min, and the flow-through was discarded. Following washes with 500 µL of Buffer AW1 (centrifuged at 8000 rpm for 1 min) and Buffer AW2 (centrifuged at 13,000 rpm for 3 min), the column was air-dried for 3 min at room temperature. DNA was eluted with 100 µL of Buffer AE onto the column membrane, incubated for 5 min at room temperature, and centrifuged at 10,000 rpm for 1 min. DNA was stored at 4 °C. All procedures adhered to laboratory standards and manufacturer’s guidelines, including biosafety measures and proper reagent handling.

### 2.4. DNA Quantification

After DNA extraction, 2 µL of each sample was used for quantification using a Nanodrop 2000 spectrophotometer (Thermo Scientific) at the Immunoparasitology Laboratory of Instituto Oswaldo Cruz. DNA quantification was performed at a wavelength of 260 nm. Before readings, samples were gently mixed, and the Nanodrop analysis cell was cleaned and equilibrated. The equipment was calibrated using ultrapure water as a blank, and the baseline was adjusted. Three independent readings were taken for each sample, and the DNA concentration was calculated using the Beer–Lambert law. Additionally, DNA purity was assessed by the 260 nm/280 nm absorbance ratio. The results were recorded, and the DNA concentration was standardized for subsequent experiments.

### 2.5. Primer Selection

Taxon-specific oligonucleotide primers were used to detect target microorganisms. A pair of universal bacterial primers targeting nearly all bacterial 16S rRNA genes at the same position served as a positive control for PCR, indicating bacterial presence in clinical samples (Table 1).

### 2.6. Polymerase Chain Reaction (PCR)

**Figure 1:**
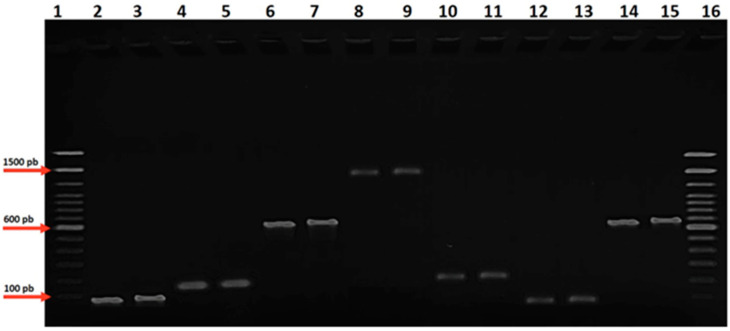
Gel electrophoresis of subgingival plaque samples, amplified by PCR and run on an agarose gel, displaying the molecular markers for bacterial species identified in our study. Lane 1 displays the molecular weight marker (100 bp). Lanes 2 and 3: bands for *P. gingivalis*. Lanes 4 and 5: *P. endodontalis*. Lanes 6 and 7 display bands for *F. alocis*. Lanes 8 and 9 display bands for *A. geminatus*. Lanes 10 and 11 display bands for *C. albicans*. Lanes 12 and 13 display bands for *T. forsythia*. Lanes 14 and 15 display bands for *S. sputigena*. Each band represents the positive amplification of the respective bacterial pathogen found in our study.

The methodology for DNA amplification by Polymerase Chain Reaction (PCR) utilized the PCR Master Mix from Promega, specifically optimized for routine PCR. Each amplification reaction was conducted in a final volume of 50 µL, comprising the following components: 25 µL of PCR Master Mix, 1.2 µL of Forward primer (FOW), 1.2 µL of Reverse primer (REV), 5 µL of DNA template, and 17.6 µL of water. The PCR Master Mix contains essential components for DNA amplification. After preparing the reaction mix, samples were subjected to a thermal cycler programmed to perform denaturation, annealing, and extension steps tailored to the specific DNA template (Table 1). Positive and negative controls were included to monitor amplification specificity and efficiency. Following PCRs, amplified products were analyzed by agarose gel electrophoresis to verify successful amplification and the presence of the expected DNA fragment size. Amplification was carried out using the Veriti Thermal Cycler (Applied Biosystems, Foster City, CA, USA). Post-PCR, products were separated on a 3% Agarose gel (Sigma Aldrich, St. Louis, MI, USA) at 4 V/cm in 1× TAE buffer (0.04 M TRIS-acetate, 1 mM EDTA). Sample loading on the gel involved preparing a mixture for the 100 bp marker, comprising 5 µL of marker and 3 µL of GelRed, and another for our sample, consisting of 2 µL of loading dye, 2 µL of GelRed, and 10 µL of amplified product. Samples were carefully loaded into the gel wells, and electrophoresis was conducted at 110 V for 50 min. Following the electrophoresis run, pre-amplified products were visualized under ultraviolet (UV) illumination to identify DNA bands resulting from amplification. The GeneRuler 100 bp Plus DNA Ladder (Thermo Scientific, Waltham, MA, USA) served as a molecular size standard to estimate the size of amplified products. Comparing bands from our sample with the ladder allowed for the approximate determination of amplified fragment sizes. The visualization of DNA bands under UV light enabled assessment of the amplification efficiency and confirmation of the expected DNA fragment size. Electrophoresis results were recorded for subsequent data analysis and interpretation (Figure 1).

### 2.7. DNA Sequencing

DNA samples, purified accordingly, were distributed into a 96-well plate (Applied Biosystems-PN. c/10 plates). For each well, a final volume of 7.5 μL was added, containing the necessary primers and Milli-Q water, as applicable. The DNA sequencing reaction mixture was calculated based on the number of samples to be analyzed per plate. For each plate, the required amount consisted of 100 μL of BigDye (Applied Biosystems-PN.:4336917-100-reaction kit) and 150 μL of 5× sequencing buffer (Applied Biosystems-PN:4336699). The plate was sealed with a PCR plate adhesive or Axygen rubber mat (AxyMat-Code CM-96-RD). Next, 2.5 μL of the reaction mixture was added to each well of the plate (1 μL of BigDye + 1.5 μL of 5× buffer). The plate was briefly centrifuged to ensure proper mixing. The following thermal cycler protocol was used: 94 °C for 10 s, 50 °C for 5 s, and 60 °C for 4 min, repeated for 40 cycles. After the sequencing reaction, samples underwent precipitation to remove free ddNTPs, which could interfere with DNA sequence reading on the DNA analyzer. The plate was briefly centrifuged, and then 30 μL (3× volume) of 75% isopropanol (MERCK) was added to each well. Samples were vortexed for 10 s, followed by a 15 min incubation period, protected from light, and centrifuged again at 4000 rpm for 45 min. The supernatant was discarded by pouring the plate over paper towels and gently tapping it on the bench. Then, 50 μL (5× volume) of 75% ethanol (MERCK) was added to each well of the plate, which was centrifuged again for 15 min at 4000 rpm. The supernatant was discarded using paper towels, and the plate was inverted and centrifuged at 600 rpm for ethanol absorption, stopping when reaching 600 rpm. Subsequently, the plate was placed on a 60 °C heat block for 10 min, protected from light. The resulting dry plate could be stored, protected from light, at −20 °C for a maximum of approximately 30 days. For the final step, 10 μL of Hi-Di formamide (Applied Biosystems P/N:4311320) was added to each well of the dry plate, which was briefly centrifuged. Then, the plate was placed on a 95 °C heat block for 3 min, protected from light, and immediately transferred to ice for 5 to 10 min. After brief centrifugation to allow the products adhered to well walls to concentrate at the bottom, the plate was subjected to capillary electrophoresis using an ABI 3730xl instrument.

### 2.8. Sequence Analysis

The genetic diversity of the 16S gene fragments was analyzed using DnaSP v6 software to estimate the diversity within the population based on genetic diversity parameters such as number of segregating sites (S), number of haplotypes (h), haplotype diversity (Hd), and nucleotide diversity (π). Natural selection on the 16S gene fragments was assessed using Tajima’s D test and the Z test. Tajima’s D values were calculated using the total number of mutations estimated with DnaSP v6 software to test the neutral theory of evolution. Positive values may suggest positive or balancing selection, maintaining alleles at balanced frequencies, while negative values indicate purifying selection or recent population expansion. The Z-test was performed with MEGA7 v6.0; non-synonymous to synonymous substitution rates (dN/dS) (1000 bootstrap replicates) were estimated using the Nei-Gojobori method with Jukes correction, where *p* < 0.05 was considered significant.

## 3. Results

### 3.1. General Epidemiological Data

A total of 24 patients participated in the study, out of which 21 completed the epidemiological data form. The mean age of the participants was 38 years, ranging from 25 to 60 years. Regarding gender distribution, 7 participants were female, 10 were male, and 4 did not provide this information.

The duration since HIV diagnosis ranged from 2 to 23 years. Participants’ educational levels varied, with two having incomplete elementary education, seven completing elementary school, two having incomplete high school education, and six completing high school. Information on educational attainment was not available for four participants.

The clinical and epidemiological characteristics of the 21 participants are summarized in Table 2. The mean age of the cohort was 42.5 years (±7.9), and the average duration since diagnosis was 8.2 years (±6.2). Hematological assessments revealed a mean leukocyte count of 4783 cells/mm^3^ (±2096), with lymphocyte and neutrophil counts averaging 1758 cells/mm^3^ (±696) and 2319 cells/mm^3^ (±1158), respectively. The immunological profile included a mean T-CD4 count of 661.4 cells/mm^3^ (±354.1) and a mean T-CD8 count of 792.9 cells/mm^3^ (±397.1), resulting in a CD4/CD8 ratio of 0.865 (±0.286).

In terms of clinical comorbidities, 17% of participants were diagnosed with diabetes, and 22% had hypertension. Additionally, 28% of the cohort experienced herpes simplex virus infections, and another 28% were diagnosed with shingles. These findings indicate a notable prevalence of both metabolic and infectious conditions within the study population, emphasizing the need for comprehensive management strategies to address these associated health issues.

Clinical and epidemiological data were collected and summarized in the following Table 2.

### 3.2. Clinical Measurements

Clinical measurements [PPD, CAL, BOP, plaque accumulation (PL), and visible supragingival biofilm] were recorded at six sites per tooth in all teeth of all patients by two trained examiners, using a conventional manual periodontal probe (Hu-Friedy, Chicago, IL, USA).

Table 3 presents a summary of the clinical parameters of all the patients studied. VSB percentages ranged from 6.8% to 90.5%, with the highest biofilm presence in patient 5 (90.5%) and the lowest in patient 14 (6.8%). BOP values varied from 2.8% to 77.7%, with the highest value found in patient 12 (77.7%) and the lowest in patient 4 (2.8%). PPD measurements ranged from 1.29 mm to 3.59 mm, with patient 24 exhibiting the deepest probing depth (3.59 mm) and patient 21 the shallowest (1.29 mm). CAL values ranged from 0.20 mm to 4.47 mm, with the highest attachment loss observed in patients 10 (4.47 mm) and 12 (3.88 mm), while patient 13 showed the lowest CAL (0.0 mm). These findings indicate considerable variability in periodontal conditions across the patients, with significant differences in biofilm accumulation, gingival inflammation, probing depth, and attachment loss.

### 3.3. Molecular Identification of Targeted Oral Pathogens

**Figure 2:**
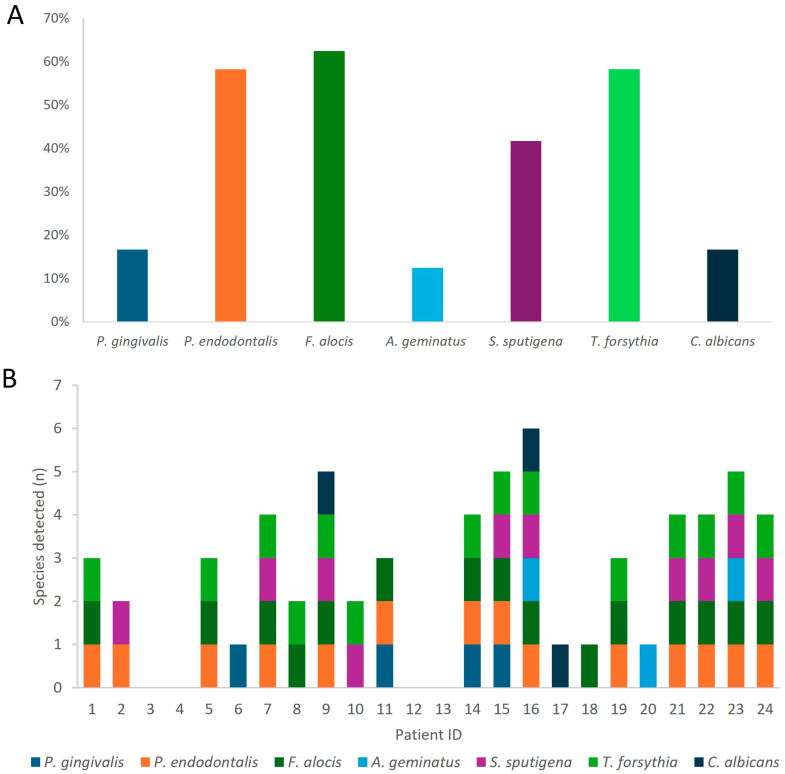
Molecular identification of oral pathogens in dental plaque samples of patients with HIV. (**A**) Frequency of PCR-detected pathogenw among all studied samples from patients with HIV (n = 24). (**B**) Interspecific diversity detected by PCR in plaque samples from each patient with HIV enrolled in the study.

In our study on the molecular identification of oral pathogens, we successfully identified the presence 7 out of the 12 targeted bacterial genes in our collected samples. These include *P. gingivalis*, *P. endodontalis*, *F. alocis*, *A. geminatus*, *C. albicans*, *T. forsythia*, and *S. sputigena* (Figure 2). Among the 24 patients from whom subgingival plaque was collected, 20 tested positive for bacterial presence in their samples (Figure 2). Specifically, four patients had single bacterial infections, three had infections with two bacteria, four had infections with three bacteria, four had infections with four bacteria, four had infections with five bacteria, and only one patient had infections with six bacteria. Combinations of *F. alocis*, *T. forsythia*, and *P. endodontalis* were the most frequently observed bacterial combinations.

The most frequently detected bacterium was *F. alocis*, found in 15 samples, representing 62.5% of the total. This was followed by *T. forsythia* and *P. endodontalis*, each identified in 14 samples (58.3%). *S. sputigena* was detected in 10 samples (41.6%), while both *P. gingivalis* and *C. albicans* were found in 4 samples each, accounting for 13.6%. *A. geminatus* was least common, found in three samples, representing 12.5%.

Our descriptive findings highlight the prevalence of putative periodontal pathogens in our study cohort, albeit below normal levels and/or undetected for some pathogens. *P. gingivalis* was detected in only 13.6% of samples, while *Tanerella forsythia* was more prevalent at 58.3%. *Treponema denticola* was not detected. *F. alocis* emerged as the most frequently detected bacterium, followed by *Tanerella forsythia*, a well-known periodontal pathogen, and *P. endodontalis*, an endodontic pathogen, which was found at a high frequency in the subgingival plaque of our HIV-positive patients.

Last, to contextualize our findings with the Brazilian clinical and epidemiological scenario, we also examined published studies on the same periodontal pathogens in non-HIV individuals (Table 4). Our results indicate small variations in the prevalence of microbial species in PLHIV compared to HIV-negative individuals from other Brazilian studies (Table 4). *P. gingivalis* was detected in 16.7% of PLHIV, a lower prevalence than HIV-negative individuals (29.4%) but still within the reported range (7.5–58.4%). *P. endodontalis* showed a high prevalence (58.3%) in HIV-positive individuals, similar to the 50.0–77.0% range in HIV-negative individuals. *F. alocis* was found in 62.5% of PLHIV, higher than the average in HIV-negative studies but within the reported range (20.0–67.0%). Conversely, *A. geminatus* was present in 12.5% of PLHIV, lower than the 33.0% reported in HIV-negative individuals. *S. sputigena* (41.7%) and *T. forsythia* (58.3%) had frequencies comparable to or slightly higher than those in HIV-negative individuals (15.4–67.0% and 12.0–100%, respectively). Finally, *C. albicans* was present in 16.7% of PLHIV, below the average for HIV-negative studies but within the reported range (14.5–50.0%). Notably, all values found in our study fell within the ranges reported by other Brazilian studies [23,24,25,26,27,28,29,30,31,32], suggesting that the oral microbiota of PLHIV follows previously described patterns. However, the wide variability in microbial frequencies across studies highlights the need for further research to clarify these comparisons and better understand the underlying factors driving these differences.

### 3.4. Intraspecific Diversity Analysis

Sequencing the PCR-amplified gene fragments of all participants revealed that the amplicon sequences of Brazilian isolates studied were 100% conserved in *P. gingivalis*, *F. alocis*, *A. geminatus*, *C. albicans,* and *T. forsythia*. However, notable intraspecific variability was observed in amplicons of *P. endodontalis* and *S. sputigena* (Supplementary File S1).

The polymorphisms in the *P. endodontalis* gene were investigated, focusing particularly on single nucleotide polymorphisms (SNPs). The strain ATCC 35406 was used as the wild-type reference sequence, and a collection of Brazilian isolates was analyzed for the presence of SNP polymorphisms. Three key SNPs were identified: G635T, G704A, and C714T. G635T and G704A showed genetic variation in Brazilian isolates, with frequencies of 17%, while C714T was present in all Brazilian isolates (100%) but absent in the ATCC 35406 reference sequence.

**Figure 3:**
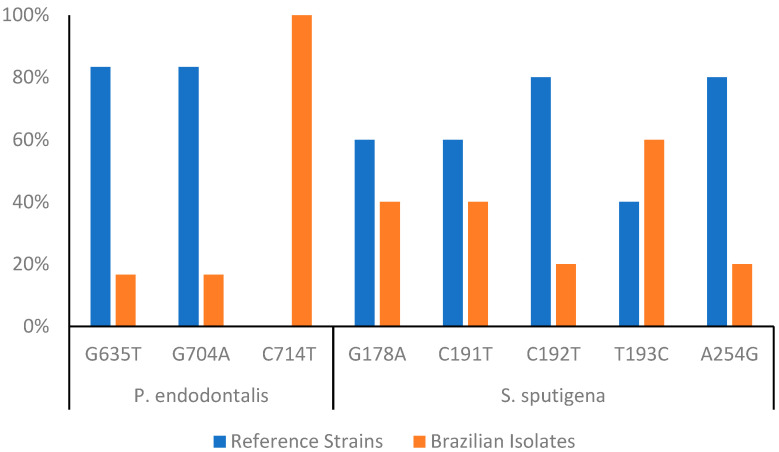
Single nucleotide polymorphism frequency in *P. endodontalis* and *S. sputigena* relative to undocumented sequences from our Brazilian cohort and the reference strains ATCC 35406 and ATCC 35185, respectively.

*S. sputigena* demonstrated notable polymorphism compared to all the bacteria identified in our study, including our previous analyses. Five key SNPs were identified in the *S. sputigena* gene, highlighting genetic variability in Brazilian isolates: G178A (40%), C191T (40%), C192T (20%), T193C (60%), and A254G (20%) (Figure 3).

## 4. Discussion

Understanding microbial diversity in individuals living with HIV is essential for assessing potential impacts on oral and systemic health. This study aimed to characterize the interspecific and intraspecific variations in microbial communities among 24 Brazilian HIV-positive individuals using molecular techniques. The decision to include this number of individuals, while it may limit certain analyses, aligns with numerous studies investigating the oral microbiota in Brazilian patients with HIV. In this scenario, we successfully identified the presence of 7 out of the 12 targeted genes, including *P. gingivalis*, *P. endodontalis*, *F. alocis*, *A. geminatus*, *C. albicans*, *T. forsythia*, and *S. sputigena* [33].

The most prevalent bacterium in our cohort was *F. alocis*, found in 15 samples (62.5%), followed by *T. forsythia* and *P. endodontalis*, each identified in 14 samples. In contrast, *P. gingivalis* and *C. albicans* were detected in only four samples each, while *A. geminatus* was the least common, present in three samples [4,34,35]. These findings suggest that the oral microbial profile of HIV-positive individuals may differ from that of the general population, potentially due to the immunocompromised state and altered host–microbe interactions in this patient population [36,37].

Interestingly, while *T. forsythia* is a well-known periodontal pathogen, *P. gingivalis* was detected at a relatively low frequency (13.6%) in our cohort, which is in contrast to its established role in the pathogenesis of periodontal disease [38]. *Treponema denticola*, another key periodontal pathogen, was not detected in any of the samples. These interesting findings may be a result of the use of HAART in our patient population, which has been shown to influence the oral microbiome [39].

The high prevalence of *F. alocis*, an emerging oral pathogen, in our study cohort is particularly noteworthy. *F. alocis* has been associated with periodontal disease and has been reported to exhibit increased abundance in HIV-positive individuals [40]. The prevalence of this bacterium, along with the high frequency of *T. forsythia* and *P. endodontalis*, suggests that the oral microbiome of HIV-positive individuals may be dominated by a distinct set of pathogens compared to the general population. However, further research is needed to fully understand the role of these bacteria in the oral health of immunocompromised individuals [41].

These findings suggest that the oral microbial profiles of HIV-positive individuals may differ significantly from those typically associated with periodontal diseases in the general population. The high prevalence of *F. alocis*, a less well-studied oral pathogen, underscores the need for further research into its potential role as an opportunistic pathogen and its impact on the oral health of immunocompromised patients [34,37].

Additionally, the relatively low detection rates of classic periodontal pathogens, such as *P. gingivalis* and *T. denticola*, highlight the complex and diverse nature of the oral microbiome in HIV-positive individuals. Moreover, the high frequency of *P. endodontalis*, an endodontic pathogen, is particularly notable, as it suggests that HIV-positive individuals may be at increased risk of endodontic infections and complications. These findings underscore the need for comprehensive oral health management strategies that address the diverse and potentially opportunistic nature of the oral microbiome in HIV-positive individuals [38]. This may be due to the unique immunological and environmental factors that shape the oral microbial ecology in this patient population, which can differ from the general population [40].

In this context, the inability to determine when patients first acquired HIV represents a limitation of our study, as this information could have provided valuable context around the time elapsed since the infection; however, the nature of our sample collection did not allow for this analysis, as none of the individuals studied could inform or estimate the date of initial infection. Moreover, a limitation of the design of our study is the absence of a group comprising HIV-negative individuals. While this study provides valuable foundational data on microbial diversity and pathogen prevalence among HIV-positive individuals, future research incorporating a control group will be critical for distinguishing the effects of HIV infection from other confounding factors, thereby advancing our understanding of oral health challenges in this population. To overcome this limitation, we accessed the frequency of the detected species in Brazilian patients without HIV infection from other studies [23,24,25,26,27,28,29,30,31,32]. This additional approach suggests that the microbial composition in PLHIV aligns with previously reported frequencies in the Brazilian population, with some species showing small variations in prevalence. While *P. gingivalis* was less frequent in PLHIV compared to HIV-negative individuals [23,24,25,26,27,28], *P. endodontalis* and *F. alocis* exhibited similar or higher detection rates. Other species, such as *A. geminatus* and *C. albicans*, appeared less prevalent in PLHIV, whereas *S. sputigena* and *T. forsythia* showed comparable or slightly increased frequencies. Despite these variations, all observed values remained within the ranges reported in other Brazilian studies. The broad variability in microbial prevalence across studies reproduce the complexity of oral microbiota dynamics and increase the need for further research to explore potential influencing factors, such as host immune status, environmental conditions, and methodological differences in microbial detection.

Regarding the interspecific diversity found in our study, the high prevalence of various putative periodontal pathogens, including *F. alocis*, *T. forsythia*, and *P. endodontalis*, emphasizes the complex nature of the oral microbiome in HIV-positive individuals. These findings have important implications for the oral health management of this patient population, as they suggest the need for targeted interventions and close monitoring to prevent the development of periodontal and endodontic complications [42]. The identification of polymorphisms that impact molecular diagnostics is a crucial and relevant aspect of sequencing 16S gene segments in oral bacterial samples. These polymorphisms represent genetic variations in DNA sequences that can influence the structure and function of molecules, including 16S segments, which are widely used as targets for taxonomic analysis and bacterial identification.

Detecting polymorphisms in 16S sequences can provide data about the evolution and phylogeny of oral bacteria. Such information is essential for understanding the relationships between different bacterial species and how they have adapted to the oral environment over time. Therefore, in the final phase of our study, we focus on sequencing the amplified DNA fragments to identify single nucleotide polymorphisms (SNPs) in the amplicons of detected species. By identifying polymorphisms that can be associated with specific diseases or relevant clinical characteristics, we aim to develop more sensitive and specific molecular diagnostic techniques. This approach could lead to more effective and personalized diagnostic tests tailored to individual patient needs. Due to a limitation in our sample and amplicon size, we did not find any significant SNPs in the majority of amplicons studied. However, in *P. endodontalis* and *S. sputigena*, we did observe some interesting SNPs. Using the *P. endodontalis* strain ATCC 35406 as a reference, Brazilian isolates were screened for SNPs. Three notable SNPs were identified in G635T and G704A. These SNPs were present in 17% of the Brazilian isolates. C714T: This SNP was present in 100% of the Brazilian isolates but absent in the ATCC 35406 reference strain, suggesting a possible distinction between the Brazilian isolates and the reference strain. Despite the limited sample size and the low number of substitution sites, these SNP findings warrant further investigation, as they may provide data into the genomic diversity and potential virulence factors of *P. endodontalis* in the context of HIV infection. Interestingly, *S. sputigena* exhibited a higher degree of polymorphism compared to other bacteria in the study. Five SNPs were identified in the *S. sputigena* gene (G178A and C191T, present in 40% of the Brazilian isolates; T193C, observed in 60% of the Brazilian isolates; and C192T and A254G, found in 20% of the Brazilian isolates). These findings corroborate the genomic diversity of a key target region for the molecular identification of *S. sputigena*, an opportunistic pathogen that has been associated with periodontal diseases. Last, while our study employed a targeted molecular approach to efficiently detect specific oral pathogens associated with periodontal and endodontic infections in HIV-positive patients, advancing to whole-genome sequencing or larger amplicon analyses in future research could provide a more comprehensive understanding of the microbiota and genetic variability. Despite the limitations of our approach, it effectively described the prevalence of key pathogens in microbial dynamics and methods for improving diagnostic strategies in this population.

## 5. Conclusions

In conclusion, our study successfully identified several oral pathogens in subgingival plaque samples from HIV-positive patients, with *F. alocis*, *T. forsythia*, and *P. endodontalis* being the most frequently detected. The molecular approach revealed a diverse range of bacterial infections, with most patients harboring multiple pathogens. Notably, *F. alocis* emerged as the predominant pathogen, and we observed a significant prevalence of *T. forsythia*, a well-established periodontal pathogen. Our findings underscore the complex microbial landscape in the oral cavity of patients with HIV, highlighting the need for further research into the interplay between these pathogens and HIV. Additionally, intraspecific diversity was evident in *P. endodontalis* and *S. sputigena*, suggesting that genetic variability in Brazilian isolates is a field to be explored with full 16S sequencing. Lastly, this study provides a reference for future studies aimed at improving molecular diagnostic and therapeutic strategies for managing oral infections in this population.

## Figures and Tables

**Table 1:** Primers and the required amplicon lengths for the analysis.

Targets	Primer Pairs (5′–3′)	Amplicon Length (bp)	Reference
* Porphyromonas gingivalis *	ACC TTA CCC GGG ATT GAA ATG	83	[12]
	CAA CCA TGC AGC ACCT AC ATA GAA		
* Porphyromonas endodontalis *	GCT GCA GCT CAA CTG TAG TCT TG	110	[12]
	TCA GTG TCA GAC GGA GCC TAG TAC		
* Filifactor alocis *	CAG GTG GTT TAA CAA GTT AGT GG	594	[13]
	CTA AGT TGT CCT TAG CTG TCT CG		
* Anaeroglobus geminatus *	GTGCTGCAGAGAGTTTGATCCTGGCTCAG	1408	[14]
	CACGGATCCTACGGGTACCTTGTTACGACTT		
* Selenomonas sputigena *	AGAGTTTGATCCTGGCTCAG	700	[15]
	CTCAATATTCTCAA GCTCGGTT		
* Enterococcus faecalis *	GTT TAT GCC GCA TGG CAT AAG AG	310	[16]
	CCG TCA GGG GAC GTT CAG		
* Treponema lecithinolyticum *	CTT GCT CCT TTC TGA GAG TGG CGG	950	[17]
	ACG CAT CCG TAT CTC TAC GAA CTT		
* Treponema médium *	AGA GTT TGA TCC TGG CTC AG	156	[17]
	CCT TAT GAA GCA CTG AGT GTA TTC		
* Fretibacterium fastidiuosum *	AGA GTT TGA TCC TGG CTC AG	969	[18]
	GAA AGT ACG TCG TCG CCC TTT CAG		
* Treponema denticola *	TAA TAC CGA ATG TGC TCA TTT ACA T	316	[19,20]
	TCA AAG AAG CAT TCC CTC TTC TTC TTA		
* Tannerella forsythia *	AGC GAT GGT AGC AAT ACC TGT C	88	[12]
	TTC GCC GGG TTA TCC CTC		
* Candida albicans *	GCC GGT GAC GAC GCT CCA AGA GCT G	158	[21,22]
	CCG TGT TCA ATT GGG TAT CTC AAG GTC		

**Table 2:** Clinical and epidemiological data from all studied patients enrolled in this study.

Summarized Data from Studied Patients (n = 24)	
*Clinical and epidemiological*	Mean ± SD
Age	42.5 ± 7.9
Time elapsed since the diagnosis (years)	8.2 ± 6.2
Leukocytes	4783 ± 2096
Linfocytes	1758 ± 696
Neutrophils	2319 ± 1158
T-CD4 cells count	661.4 ± 354.1
T-CD8 cells count	792.9 ± 397.1
CD4/CD8 ratio	0.865 ± 0.286
***Co-morbities***	**Frequency (%)**
Diabetes	17%
Hipertension	22%
Herpes	28%
Zoster	28%

**Table 3:** Summary of oral clinical measurements in studied patients. VSB: visible supragingival biofilm. BOP: bleeding on probing. PPD: periodontal probing depth. CAL: clinical attachment loss. SD: standard deviation.

Patient	% VSB	% BOP	PPD (mm)				CAL (mm)			
			Mean	SD	Median	Range	Mean	SD	Median	Range
1	18.8	26.8	2.58	0.74	3.0	1.0–5.0	2.66	0.73	3.0	1.0–5.0
2	38.2	12.0	1.87	0.65	2.0	1.0–3.0	1.87	0.68	2.0	0.0–3.0
3	-	-	-	-	-	-	-	-	-	-
4	42.7	2.8	2.10	0.32	2.0	1.0–3.0	2.22	0.50	2.0	1.0–4.0
5	90.5	19.3	2.61	0.74	3.0	1.0–6.0	2.68	0.71	3.0	1.0–6.0
6	-	-	-	-	-	-	-	-	-	-
7	48.2	28.6	2.40	0.70	2.0	1.0–6.0	1.29	1.94	0.0	0.0–6.0
8	-	-	2.50	0.72	2.0	1.0–5.0	0.0	0.00	0.0	0.0–0.0
9	37.5	42.1	2.39	0.75	2.0	1.0–4.0	0.20	0.73	0.0	0.0–3.0
10	65.3	26.0	3.59	1.73	3.0	2.0–9.0	4.47	2.00	4.0	2.0–10
11	69.4	49.5	2.89	0.73	3.0	2.0–6.0	3.01	2.22	4.0	0.0–7.0
12	84.4	77.7	3.40	1.30	3.0	2.0–8.0	3.88	2.43	5.0	0.0–9.0
13	15.1	49.2	1.91	1.99	2.0	1.0–3.0	0.59	0.85	0.0	0.0–4.0
14	6.8	3.4	1.69	0.70	2.0	1.0–4.0	0.94	1.12	0.0	0.0–4.0
15	52.2	37.3	2.00	0.84	2.0	1.0–6.0	2.14	1.32	2.0	0.0–7.0
16	-	-	-	-	-	-	-	-	-	-
17	67.5	23.3	2.48	0.71	2.0	1.0–5.0	0.75	1.35	0.0	0.0–5.0
18	38.0	14.7	2.65	1.25	2.0	1.0–7.0	3.05	1.42	3.0	1.0–8.0
19	36.6	13.9	2.23	0.69	2.0	1.0–4.0	2.78	0.82	3.0	1.0–5.0
20	25.8	40.2	1.79	0.46	2.0	1.0–3.0	2.05	0.87	2.0	1.0–8.0
21	-	-	1.29	0.80	1.0	0.0–5.0	1.68	1.47	1.0	0.0–8.0
22	46.4	12.7	3.26	1.52	3.0	1.0–9.0	3.38	1.53	3.0	1.0–10.0
23	-	-	-	-	-	-	-	-	-	-
24	70.8	37.5	2.71	0.92	3.0	1.0–5.0	2.91	1.11	3.0	1.0–7.0

**Table 4:** Prevalence of microbial species in studied PLHIV and non-HIV studied individuals from Brazilian studies.

Species	HIV (+) Studied Individuals	HIV (−) Individuals from Other Brazilian Studies	Refs.
	Frequency	Mean Frequency (Range)	
*P. gingivalis*	16.7%	29.4% (7.5–58.4%)	[23,24,25,26,27,28]
*P. endodontalis*	58.3%	63.5% (50.0–77.0%)	[29]
*F. alocis*	62.5%	43.0% (20.0–67.0%)	[23,29,30,31]
*A. geminatus*	12.5%	33.0% (33.0%)	[27,31]
*S. sputigena*	41.7%	44.6% (15.4–67.0%)	[31]
*T. forsythia*	58.3%	46.2% (12.0–100%)	[24,26,28]
*C. albicans*	16.7%	27.5% (14.5–50.0%)	[32]

## Data Availability

The datasets supporting the conclusions of this article are included within the article.

## Supplementary Materials

The following supporting information can be downloaded at: https://www.mdpi.com/article/10.3390/microorganisms13040867/s1, Supplementary File S1.
